# Cultural heritage and sports tourism: a systematic literature review of sustainable destination management practices

**DOI:** 10.3389/fspor.2025.1680229

**Published:** 2025-10-15

**Authors:** Yoki Afriandy Rangkuti, Arti Kurniaty Bangun, Rizkei Kurniawan, Zulpikar Ilham, Sanusi Hasibuan, Try Marta Br Tambunan, Daniel Fransisco Silitonga

**Affiliations:** ^1^ Department of Physical Education, Faculty of Teacher Training and Education, Universitas Samudra, Langsa City, Indonesia; ^2^ Graduate Program in Sports Education, Faculty of Sports Science, Universitas Negeri Semarang, Semarang City, Indonesia; ^3^ Graduate Program in Sports Education, Faculty of Sports Science, Universitas Negeri Medan, Medan City, Indonesia

**Keywords:** cultural heritage, sports tourism, sustainable destination management, community participation, digital innovation, triple bottom line

## Abstract

This systematic literature review explores the integration of cultural heritage with sports tourism as a promising strategy for sustainable destination management between January 2020 and June 2025. Utilizing the Scopus database and reported following PRISMA 2020, this study synthesized 63 Scopus-indexed peer-reviewed journal articles to examine strategies, benefits, challenges, and governance models. Analysis highlighted commonly reported integration methods, including community-driven thematic events, educational programs, and digital innovations such as virtual and augmented reality technologies. The findings from the included studies indicate reported socio-cultural and economic benefits, such as community empowerment, infrastructure enhancement, and tourism revenue growth. However, challenges like over-commercialization, authenticity dilution, and environmental degradation remain prevalent. Effective governance emerged as crucial for sustainability, across the reviewed literature**,** emphasizing participatory and collaborative models aligned with Triple Bottom Line and Stakeholder Theories. The study contributes by offering a nuanced understanding of pathways through which cultural heritage sports tourism may support economic prosperity, cultural integrity, and environmental sustainability in specific contexts. Future research directions are recommended, including multi-database, preregistered reviews and longitudinal, regionally comparative analyses, to further validate these findings and address identified limitations comprehensively. This review is limited by a single-database (Scopus-only) search; findings should be interpreted as an exploratory synthesis.

## 1 Introduction

Sports tourism has emerged as one of the most dynamic segments within the global tourism industry, experiencing significant growth and diversification from 2000 to 2025. Initially focused predominantly around major sporting events, the field has progressively expanded to encompass active, experiential travel and integrated cultural experiences (1, 2). From 2020 to 2025, sports tourism has undergone significant changes, primarily influenced by the global COVID-19 pandemic, which initially led to a decline in tourism activities. Research by Ito and Higham indicates that the recovery strategy for sports tourism involves integrating supplemental activities to enhance the tourism experience, highlighting the importance of a comprehensive understanding of sport tourism impacts (3). By capitalizing on traditional sports and cultural events (4), many regions, such as Jayapura in Indonesia, have started to revitalize their economies, creating job opportunities and promoting infrastructure development, as noted by Guntoro et al. (5). Additionally, sports tourism is increasingly recognized as a critical component in promoting sustainable tourism, contributing not only to economic growth but also to the well-being and cultural engagement of communities (6). The trend of health-conscious tourists post-pandemic has further driven growth in the sector, leading to an uptick in participation in various sporting events and recreational activities, thus enhancing both the economic and social fabrics of host locations, as supported by Lin et al. (64) and Satiadji et al. (7). In conclusion, the global evolution of sports tourism from 2020 to 2025 reflects a shift towards sustainability, community engagement, and the leveraging of cultural heritage alongside sporting events to foster economic resilience.

Integrating sports tourism with cultural heritage is therefore a strategic imperative for sustainable destination management. Effective integration supports balanced economic growth, environmental preservation, and socio-cultural sustainability, aligning with contemporary expectations of responsible tourism practices (8, 9). The critical challenge lies in managing the delicate balance between commercial exploitation and preservation of authenticity, requiring nuanced strategies to mitigate negative socio-cultural and environmental impacts (10).

Parallel to this growth, cultural heritage tourism has also gained prominence, increasingly recognized as an essential asset in the global tourism industry. Cultural heritage serves as both an economic catalyst and a vital means of preserving and promoting local identities traditions, and historical significance (11). Integrating sports tourism with cultural heritage thus emerges as a strategic opportunity to enhance destination appeal and sustainability. This integration not only enriches the tourist experience but also fosters community pride and facilitates the preservation and revitalization of cultural heritage sites (12, 13), while explicitly engaging with theoretical debates on *staged authenticity* and *commodification* that shape how heritage is presented and consumed.

The overarching objective of this systematic literature review is to critically examine existing research that bridges sports tourism and cultural heritage. Specifically, this review seeks to identify sustainable destination management practices that leverage cultural heritage assets. By systematically analyzing the integration of these two dynamic tourism sectors, this study aims to highlight effective strategies and identify potential areas for improvement. We undertake this review because existing syntheses often treat sports tourism and cultural heritage in isolation or address governance and digital innovation superficially; our contribution is to integrate sustainability (Triple Bottom Line), stakeholder governance, and authenticity–commercialization debates within a quality-weighted synthesis and mapped geographic coverage. The review covers literature published between January 2020 and June 2025, analyzing 63 Scopus-indexed studies, encompassing a broad global perspective and a diverse array of subtopics, including sustainability, destination management, and community involvement.

For clarity, we use concise operational definitions. “Cultural heritage” denotes tangible and intangible assets (sites, practices, and knowledge). “Sports tourism” denotes travel to participate in or spectate sport, including recreation and adventure. “Sustainable destination management” refers to strategies that balance economic, environmental, and socio-cultural outcomes (Triple Bottom Line) to ensure long-term resilience.

Structurally, this review progresses through clearly delineated sections. Following this introduction, the methods section outlines the systematic approach employed, including search strategies, inclusion and exclusion criteria, and quality assessment tools, as well as inter-rater reliability and risk-of-bias procedures. The theoretical framework section reviews prominent theories and historical perspectives relevant to sports tourism and cultural heritage integration, linking authenticity–commercialization debates to the analytical lenses used. Subsequently, thematic findings are systematically presented, covering integration methods, sustainability practices, socio-cultural impacts, economic implications, digital innovation, and governance frameworks, alongside a geographical distribution of the included studies and a summary of study quality appraisal. The review concludes with a critical discussion synthesizing key findings, highlighting strategic recommendations, and identifying future research directions.

In summary, this systematic literature review addresses a timely and significant intersection within the global tourism sector, emphasizing the importance of integrating sports tourism and cultural heritage for sustainable destination management. By thoroughly examining scholarly contributions from January 2020 to June 2025, this study aims to provide valuable insights and practical guidance for tourism stakeholders, policymakers, and researchers dedicated to fostering sustainable, culturally enriching tourism development. Given the Scopus-only search, findings are presented as an exploratory synthesis with transparent limitations.

## 2 Methods

### 2.1 Search strategy

The systematic literature review (SLR) utilized a structured search strategy designed to comprehensively identify relevant scholarly articles addressing the integration of sports tourism with cultural heritage for sustainable destination management. The protocol was pre-registered on OSF (registration link/DOI redacted for peer review) and search conduct/reporting followed PRISMA 2020 guidance. The primary database employed was Scopus, selected for its extensive coverage of peer-reviewed literature within tourism and related fields. A multi-block search strategy combined heritage term*s (“cultural heritage” OR “intangible cultural heritage” OR museum OR heritage*), sport-tourism terms (“sport* tourism” OR “sports tourism” OR “event tourism” OR marathon OR cycling OR “traditional sport*” OR “indigenous game*”), and sustainability/management terms (sustainab* OR “destination management” OR governance OR “carrying capacity” OR “triple bottom line”), using TITLE-ABS-KEY fields and Boolean AND; full search strings are provided in Supplementary Appendix A. We limited results to English, peer-reviewed journal articles published between January 2020 and June 2025 and conducted backward/forward citation chasing to mitigate database bias. Use of Scopus alone is a study limitation and is acknowledged in the Discussion.

### 2.2 Inclusion and exclusion criteria

Clearly defined inclusion and exclusion criteria were established to enhance the rigor and precision of this systematic review, and were applied independently by two reviewers with disagreements resolved by a third adjudicator.

#### 2.2.1 Inclusion criteria

The literature included met the following requirements.

• Peer-reviewed journal articles.
• Published in the English language.
• Clearly focused on the integration of sports tourism with cultural heritage.
• Published between January 2020 and June 2025, to ensure currency and relevance.
• Full-text available.
• Indexed in Scopus at the time of search.

Articles included provided empirical, conceptual, or methodological insights into sustainable destination management practices, specifically those leveraging cultural heritage within the realm of sports tourism. Consistent with a quality-weighted synthesis, studies were retained irrespective of design provided methodological clarity allowed appraisal**.** Examples of included studies are Du et al.'s (12) exploration of the Tulou World Heritage marathon event and its impact on tourism loyalty, and Husain et al.'s (14) use of multi-criteria decision analysis for sustainable planning in Indonesia.

#### 2.2.2 Exclusion criteria

Studies were excluded based on the following criteria

• Conference papers, book chapters, and grey literature.
• Non-English articles.
• Articles published before 2020.
• Research that did not explicitly address both sports tourism and cultural heritage in the context of sustainable management.
• Duplicates identified across search rounds.
• Studies lacking sufficient methodological detail for quality appraisal.

For example, purely theoretical discussions without clear relevance to the integration of sports and cultural heritage, or studies focused solely on economic outcomes without addressing sustainability, were excluded to maintain clarity and focus.

### 2.3 Screening and selection process

#### 2.3.1 Initial screening

Two reviewers independently screened titles/abstracts, followed by full-text assessment of potentially eligible records. Disagreements were resolved through discussion; where consensus could not be reached, a third reviewer adjudicated. Inter-rater reliability targets were set at Cohen's *κ* ≥ 0.70 for both screening stages.

A screening log documented decision rationales (include/exclude) and coded reasons for exclusion (e.g., focus on mega-events, non-tourism context, insufficient methods). This protocol ensures reproducibility and auditability.

#### 2.3.2 Full-text screening

Following the preliminary screening, full texts of remaining articles were reviewed in detail to confirm their eligibility. Two reviewers independently assessed all full texts; Cohen's *κ* was calculated, and reasons for exclusion at this stage were recorded and are provided in Supplementary Appendix B. The full-text review process further refined the selection, ensuring adherence to the inclusion criteria and relevance to the research objectives. Articles that did not fulfill these criteria were excluded at this stage.

#### 2.3.3 Quality assessment

The quality of the included articles was rigorously assessed using design-appropriate tools (Mixed Methods Appraisal Tool—MMAT 2018/2022—and relevant JBI checklists), applied independently by two reviewers with consensus resolution. PRISMA 2020 was used to guide transparent reporting, not to appraise study quality. Per-study appraisal results are presented in a dedicated Quality Appraisal Table (Supplementary Table SQA), and a sensitivity analysis considered the influence of lower-quality studies on thematic conclusions. Potential publication/reporting bias was assessed qualitatively; quantitative meta-analysis was not attempted due to heterogeneity of designs and outcomes.

### 2.4 Data extraction and analysis

Data from selected articles were systematically extracted using a predefined codebook mapping constructs to the Triple Bottom Line and Stakeholder Theory (e.g., context, heritage type, sport/event type, governance mechanisms, outcomes/indicators, and safeguards). Studies were geo-coded by country and UN subregion. The extracted information was organized into structured tables to facilitate comparative analysis and synthesis of findings (Tables 1–6), alongside a dedicated quality-appraisal table; a geographic distribution figure summarizes regional representation. Analytically, we conducted thematic synthesis with effect-direction vote counting; no quantitative meta-analysis was performed due to heterogeneity. Each table provides a succinct summary of key information, enabling clarity and ease of interpretation.

**Figure 1:**
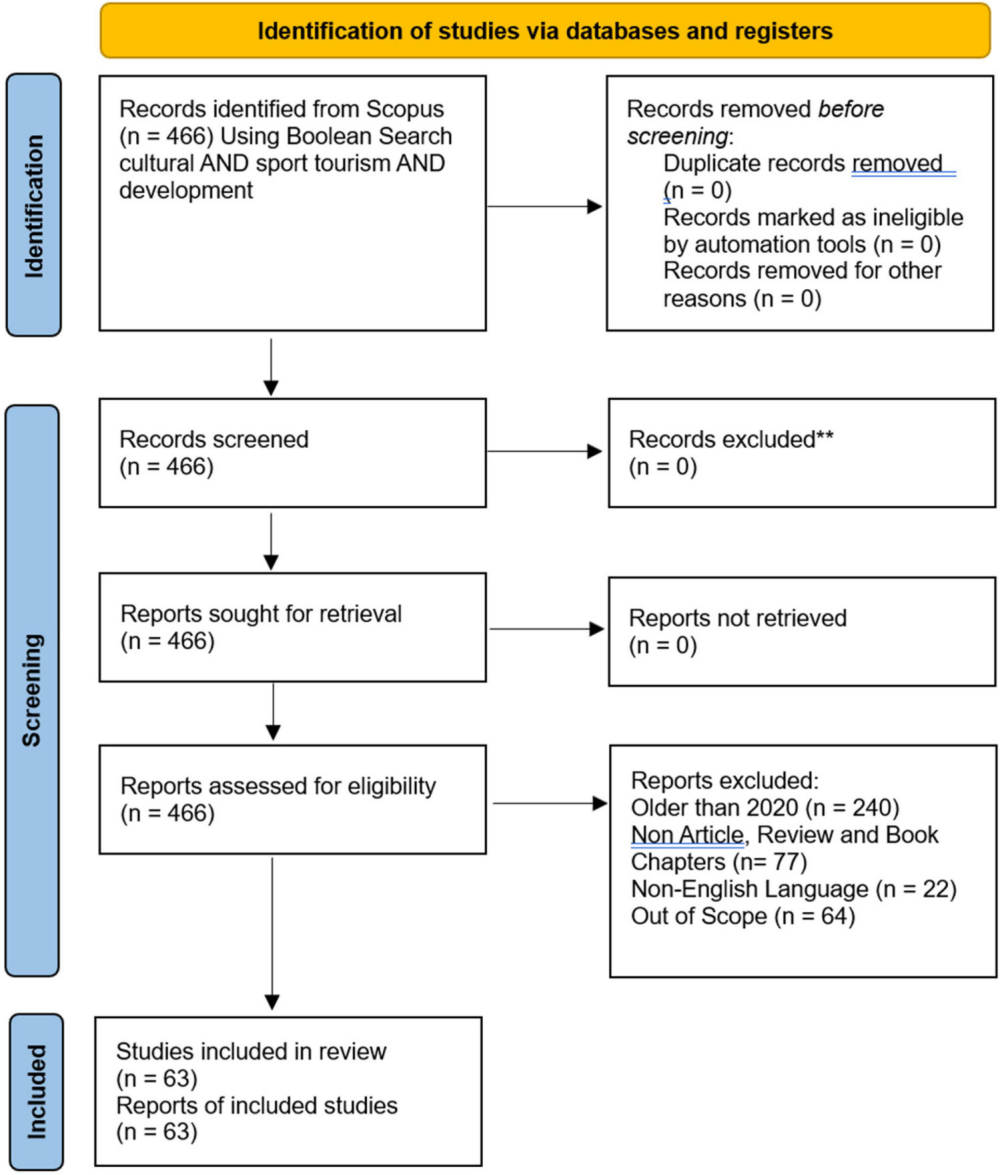
PRISMA 2020 flow diagram.

**Table 1:** Theme A—integration & governance, condensed.

No	Author(s) & Year	Cultural heritage aspects	Integration methods	Effectiveness (headline finding)	Key challenges
1	Wang et al. (15)	Chinese Wushu heritage	IoT-enabled smart experiences; event co-branding	Boosted visitor appeal & local revenue	Tech infrastructure gaps
2	Li et al. (16)	Folklore sports along Yellow River	Resident–tourist emotional solidarity model	Enhanced identity & sustainable rural tourism	Balancing resident & tourist expectations
3	Amar et al. (17)	Taji Tuta martial ritual	SWOT-led destination positioning	Identified unique market niche	HR capacity deficits
4	Yangutova et al. (18)	Siberian ski-landscape traditions	Competitiveness index for winter heritage resorts	Flagged Sobolinaya as prime hub	Uneven infrastructure investment
5	Gonzalez de la Fuente (19)	Karate heritage in Okinawa	Institutionalisation of “karate tourism”	Diversifies local economy	Tension with mainland narratives
6	Kurowska et al. (20)	Historic quarries & forest sport trails	Landscape restoration + sport routes	Reduced pressure on core sites	Need for multi-agency coordination
7	Ostrowska-Tryzno and Pawlikowska-Piechotka (21)	UNESCO sports architecture	Re-designing visitor flows post-COVID	Maintained cultural magnetism safely	Sanitary compliance costs
8	Du et al. (12)	Tulou World Heritage marathon	Event-heritage image coupling	Raised tourist loyalty	Place-attachment still weak
9	Despotovic and Koch (22)	Alpine heritage landscapes	Spatial econometrics on land value vs. ski culture	Showed heritage premium	Housing affordability trade-offs
10	Echeverri et al. (23)	Colombian biocultural richness	Integrated biodiversity-culture mapping	Identified untapped biocultural destinations	Accessibility gaps
11	Rangkuti et al. (24)	Traditional horse racing; triathlon	triathlon route design showcasing landscape & culture;	Tourist arrivals increased; stronger sustainable image	monitoring; authenticity risks
21	de Freitas et al. (25)	Portuguese medieval castles	Quantile regressions on tourism flows	Castles extend visitor stay	Need holistic rural packages

**Table 2:** Theme B—sustainability & impacts, condensed.

No	Author(s) & year	Destination/region	Sustainability measures	Outcomes	Limitations
1	Husain et al. (14)	Indonesia (generic)	MCDA model for smart sustainable planning	Balanced quality–environment targets	Complex stakeholder buy-in
2	Jiang et al. (26)	Jianmen Shu Road	TES index (DPSIR)	Spatial hotspots for protection	Data intensity
3	Tai et al. (27)	Yangtze River Delta	ESDA + grey correlation for resource optimisation	Shift from “policy-sports” to “total-factor” drive	Regional disparities
4	Stojanović et al. (28)	Kraljevac Reserve	Prism of Sustainability survey	Natural & sociocultural factors raise satisfaction	Sample limited to one reserve
5	Zhensikbayeva et al. (29)	South Altai	GIS-based resource visualisation	Proposed thematic routes	Mountain data paucity
6	Hallmann & Zehrer (65)	Various alpine sites	Sport-scape–landscape linkage	Spatial sports elevate place identity	Small N interviews
7	Boroujerdi et al. (9)	Zrebar Lake, Iran	MICMAC on six critical factors	Guides authority focus on management	Emerging-market constraints
8	Fu & Liang (68)	Island tourism development sites	SWOT sustainability audit	Highlighted unique marine appeal	Pre-COVID baseline
9	Kurowska et al. (20)	Ślęża Massif	Forest quarry re-purposing	Reduces pressure on main trails	Restoration funding
10	He and Wu (30)	Zhejiang, China	Top-5 environmental indicators ranking	Ordered environments key for eco-sports	Limited to survey perception
11	Hu et al. (13)	China (macro)	IoT-based coupling analysis	Positive sport–tourism synergy	Needs longitudinal validation

**Table 3:** Theme C—socio-cultural & participation, condensed.

No	Author(s) & year	Engagement strategy	Positive impacts	Negative impacts	Recommendations
1	Marin-Pantelescu et al. (31)	Focus-groups with Erasmus students	Enhanced host–guest understanding	Urban stress reported	Improve city liveability
2	Li et al. (16)	Emotional solidarity surveys	Stronger folklore identity	Divergent resident vs. tourist views	Tailored engagement programs
3	Pambudi and Hariandi (32)	Multi-stakeholder interviews Tour de Ijen	Economic uplift, social pride	Waste management strain	Integrate environmental education
4	Mair et al., 2023	Narrative SLR of mega-events	Framework for social benefits	Measurement inconsistency	Standardised metrics
5	Widianingsih et al.Pattaray et al. (10)	Digital trend analysis (F1H2O)	Regional promotion online	Event prep issues	HR upskilling
6	Pattaray (33)	FGDs around MotoGP	HR development model	Cultural commodification risk	Inclusive capacity building
7	Stojanović et al. (28)	Resident & visitor surveys	High satisfaction with nature & culture	Environmental concern minimal	Sustainability communication
8	Lestari and Yusra (34)	Ethnographic mapping Sasak practices	New ethno-attractions listed	Authenticity dilution fear	Community-led curation
9	Wen (35)	Spatial diffusion of ethnic sports	Cultural landscape integration	Over-commercialisation	Balanced cultural zoning
10	Komaini et al. (36)	Village-level participatory study	Sports tourism boosts local economy	Stakeholder coordination gaps	Cross-sector collaboration
11	Usmanova et al. (37)	Free tourism zones concept	Unlock heritage potential	Low tourist flow baseline	Infrastructure & marketing push

**Table 4:** Theme B—sustainability & impacts, condensed.

No	Author(s) & year	Event/activity	Economic metrics	Positive impacts	Negative impacts
1	Pambudi and Hariandi, (32)	Tour de Banyuwangi Ijen	Ticket sales ↑ 100%; SMEs revenue +43%	Reduced unemployment	Waste & crowding
2	Despotovic and Koch (22)	Alpine land price model	Spatial Durbin model – ski premium	Increased land value	Housing affordability
3	Chang et al. (38)	Sport tourism dependency	PLS SEM support factors	Resident support for sports projects	Dependency risk
4	Lohana et al. (39)	Mediation moderation model	SEM linking environment/culture & economy	GDP growth driven by sports tourism	Destination image not moderating
5	Sarmento and Monteiro (40)	Tarrafal hub workshops	Stakeholder SWOT	Diversification of local economy	Infrastructure upgrades needed
6	Sezerel and Karagoz (41)	Datça SPA surveys	Economic vs. environmental exchange	Local economic support	Environmental impacts undervalued
7	Dirin et al. (42)	Todzhinsky district GIS	Tourism potential mapping	Investment clustering identified	Limited access routes
8	Zhang et al. (60)	West Sichuan integration index	TOPSIS rankings	Education investments increased	Inter-regional inequalities
9	Offenhenden and Soronellas (43)	Pyrenees ski vs. farming	Economic case study	Rural income supplemented	Agriculture marginalization
10	Ma et al. (69)	Sports fitness rural tourism	Governance & satisfaction scores	Economic & environmental gains	Overuse risks

**Table 5:** Theme C—digital & innovation, condensed.

**No**	**Author(s) &** y**ear**	**Technology/**i**nnovation**	**Implementation** s**trategy**	**Outcomes**	**Challenges**
1	Wang et al. (15)	IoT for Wushu tourism	Smart monitoring & storytelling	Enhanced engagement	Digital divide
2	Cao & Xiao (66)	AI big-data image management	UGC analysis along BRI	Improved branding	Data privacy
3	Qiu et al. (67)	Live-stream tourism	Content analysis 48k posts	Positive emotions dominate	Illegal content risk
4	Sun et al. (1)	Fuzzy analysis algorithm	7-dim competitiveness model	Better industry benchmarking	Model complexity
5	Zhang and Ala (44)	Ontology & NER for ICH	Digital cataloguing	Supports preservation	Needs continual updates
6	Hao et al. (45)	Big-data sentiment of rural sports tourists	CF-tree clustering	Identified gender & age patterns	Short trip dominance
7	Hao et al., (46)	AI-enabled coupling model	Invisible statistical logic	New sport–culture interface	Incomplete data
8	Hu et al. (13)	IoT grey relational analysis	Coupling sport & tourism sectors	Strong correlation	Scalability
9	Ieong, 2024	SWOT for Macao study tours	Leverage National Games hype	Opens diversification	Policy framework gaps
10	Wu et al. (47)	Night-time tourism analytics	Athlete-centric cultural offers	Strategic growth roadmap	Competition for attention
11	Gharibzadeh et al. (48)	Grounded theory on sports tours	Qualitative coding	Facilitators vs. inhibitors mapped	Financial & security barriers

**Table 6:** Theme A—integration & governance, condensed.

No	Author(s) & year	Policy framework	Governance model	Strengths	Limitations
1	Tang et al. (49)	Sports–Culture–Tourism integration index	Event venue evaluation	Reliable metrics	Single case validation
2	Hu Q. et al. (50)	Spatial ICH protection model	Geo detector-based	Early identification of drivers	Eastern bias
3	Sezerel and Karagoz (41)	Special Protected Area planning	Mixed-method resident input	Validation of SET/TBL	Environmental impacts undervalued
4	Usmanova et al. (37)	Free Tourism Zones law	National strategic zoning	Unlocks heritage potential	Limited access infrastructure
5	Kaur et al. (70)	Fuzzy LP marketing allocation	State budgeting	Maximizes ROI	Hypothetical data
6	Akhundova (51)	Festival development strategy	Functional analysis	Strengthens regional branding	Generalized data
7	Mazza (8)	Strategic communication model	Stakeholder engagement	Practical behavioral change	Empirical validation needed
8	Li L. (52)	Cross-regional value transmission	Philosophical synthesis	Innovative conceptual pathways	Abstract, lacks concrete metrics

To illustrate the research process clearly, (Figure 1) presents the PRISMA 2020 flow diagram. This diagram delineates the stages of article identification, screening, eligibility assessment, and final selection, with counts at each stage and reasons for full-text exclusions provided in Supplementary Appendix B; inter-rater reliability statistics for screening are reported in Supplementary Table S0. In summary, the methodology is reported in accordance with PRISMA 2020, pre-registered on OSF, and incorporates dual independent screening with inter-rater reliability and design-appropriate quality appraisal. Given the Scopus-only database, this review should be interpreted as an exploratory synthesis with acknowledged limitations.

**Figure 2:**
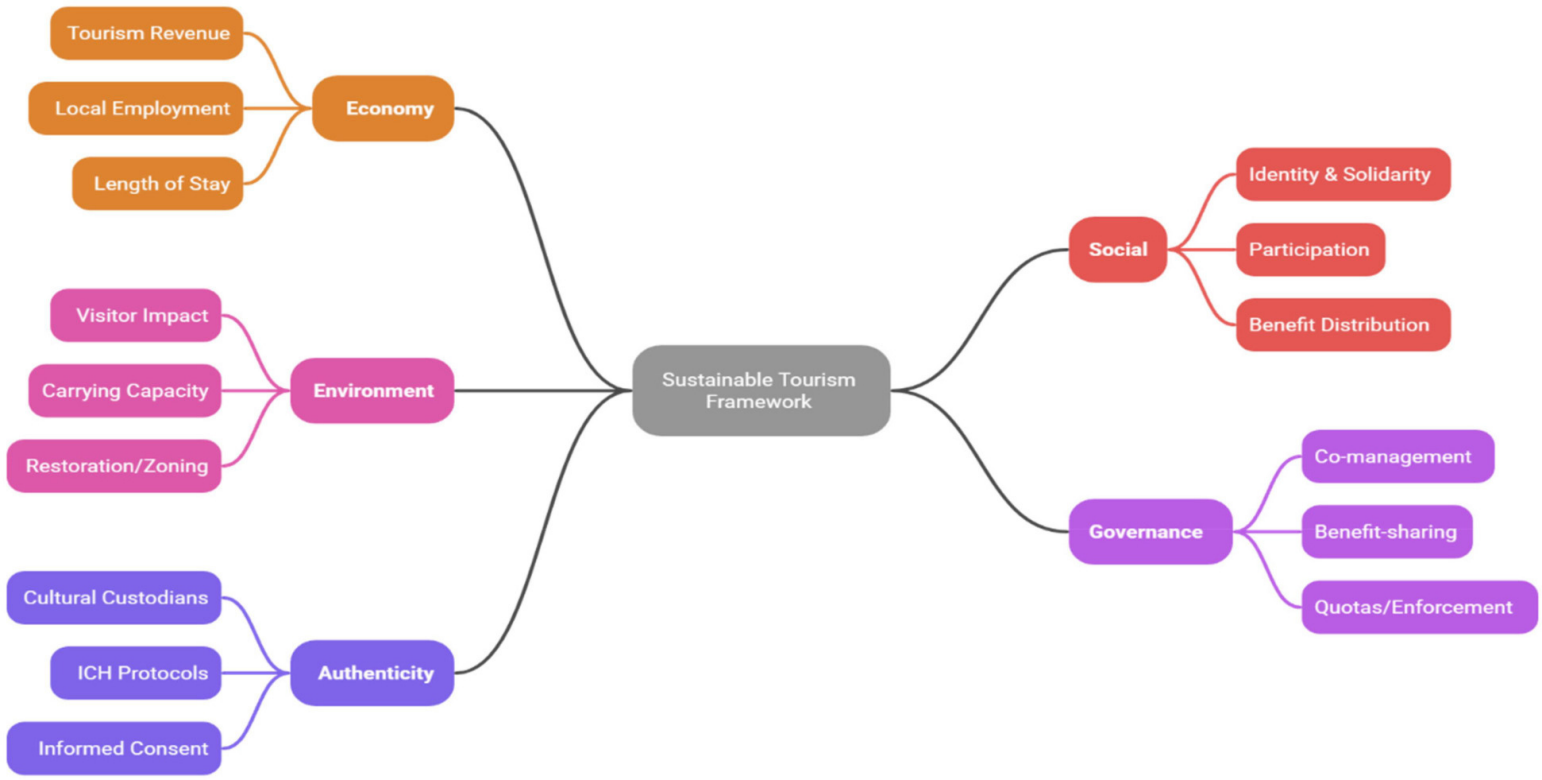
Sustainable tourism framework.

## 3 Theoretical framework

### 3.1 Relevant theories and models

The theoretical underpinning of sustainable sports tourism development integrates several models critical to understanding the interconnections between tourism, sustainability, and community engagement. Central among these frameworks are the Sustainable Tourism Development Theory, the Triple Bottom Line Model (TBL), and Stakeholder Theory, each contributing essential insights into sustainable destination management practices additionally, we draw on debates around *staged authenticity* and *commodification* to interpret tensions between experience design and heritage integrity. In this review, these frameworks are not only described but operationalized to guide data extraction and synthesis (e.g., coding outcomes by TBL domains and classifying governance/participation mechanisms under Stakeholder Theory). Figure 2 presents the operational integration model connecting these lenses and their hypothesized linkages to observed outcomes.

**Figure 3:**
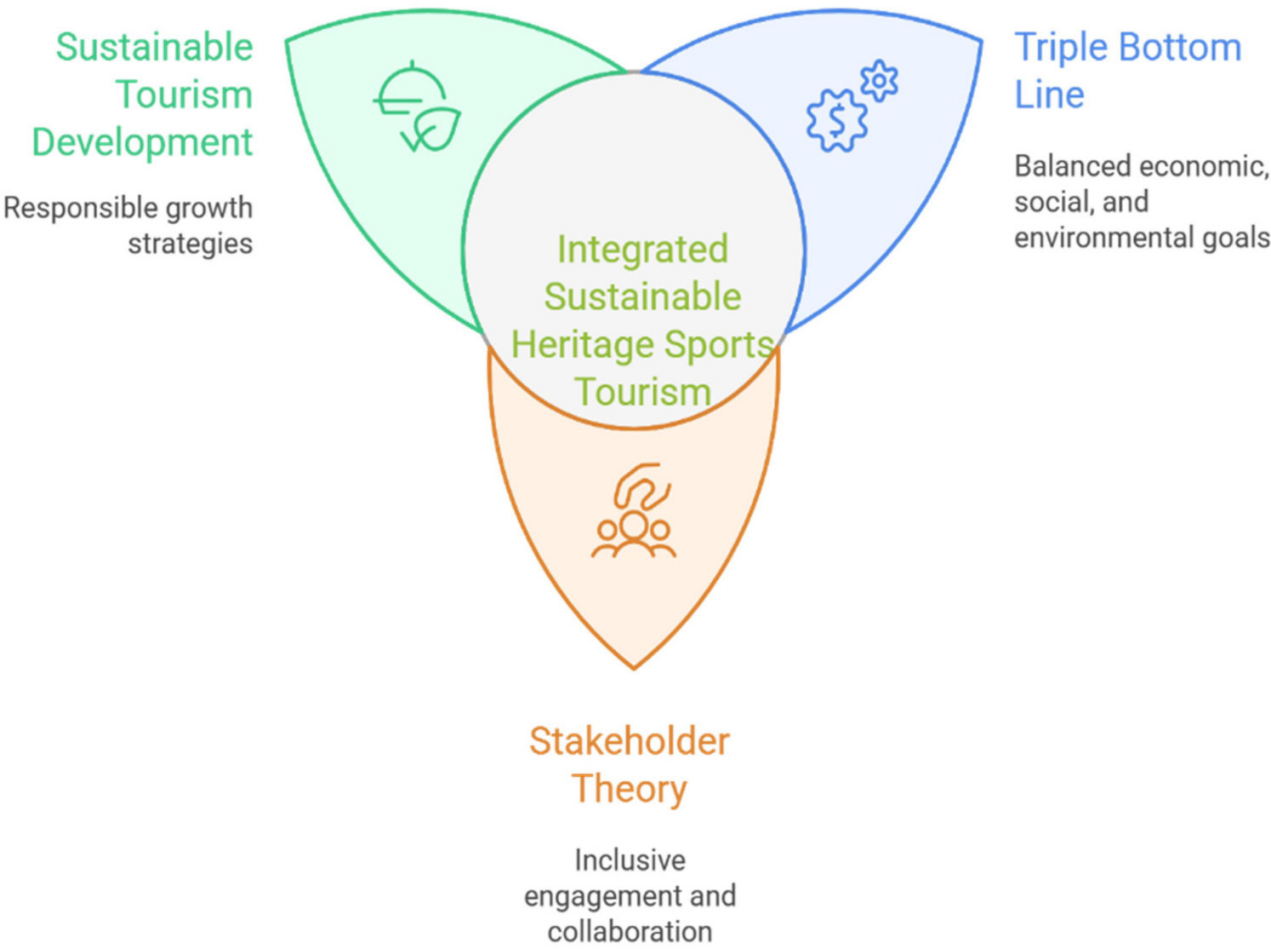
Theoretical integration model for sustainable heritage sports tourism.

Sustainable Tourism Development Theory positions tourism as a system dependent on balancing economic, environmental, and socio-cultural aspects. According to Morfoulaki et al. (2), sports tourism significantly contributes to regional economies and enhances active lifestyles while potentially risking environmental and social disruptions. Thus, sustainable development principles are integral to managing sports tourism effectively, ensuring that economic gains do not compromise ecological stability and social integrity in our analysis, this theory guided coding of contextual moderators (e.g., site protection status, event scale) and interpretation of trade-offs between access, conservation, and community wellbeing.

The Triple Bottom Line Model expands this view by explicitly considering economic prosperity, environmental quality, and social equity. Boroujerdi et al. (9) emphasize the model's utility in evaluating the sustainability of tourism projects, underscoring the necessity to maintain equilibrium among these three critical dimensions. Within heritage sports tourism, economic prosperity relates to local job creation and revenue generation, environmental quality pertains to managing visitor impacts on natural landscapes, and social equity involves preserving cultural authenticity and enhancing community livelihoods (13). In this review, we operationalized TBL as coding categories (economic, environmental, socio-cultural) and summarized effect-directions within each domain to move beyond description toward comparative synthesis.

Stakeholder Theory complements these frameworks by addressing the multifaceted interests of diverse tourism stakeholders, including local communities, visitors, governmental bodies, and commercial entities (8, 53). argues for active stakeholder engagement to achieve sustainable outcomes in tourism development, highlighting the importance of transparent communication and participatory decision-making processes. Stakeholder engagement is pivotal in resolving potential conflicts and fostering sustainable development practices aligned with community values and heritage preservation analytically, we coded governance arrangements (e.g., partnerships, co-management, community-led models) and noted practical implementation challenges (coordination capacity, resource constraints, legitimacy) to link participation structures with observed outcomes.

#### 3.1.1 Historical perspectives

The evolution of sports tourism provides critical context for contemporary practices and theoretical approaches we condense this background to foreground analytical relevance. From ancient gatherings (e.g., the Olympic tradition) to modern mega-events (54), growth has been enabled by transport development and global connectivity, while heritage tourism has been shaped by preservation movements and international recognition (e.g., UNESCO frameworks). Recent digital tools (e.g., virtual/augmented reality) broaden access and mediate heritage experiences. Here we focus on how these trajectories inform current governance and sustainability trade-offs.

#### 3.1.2 Major scholars and schools of thought

Integration of sports tourism with cultural heritage is shaped significantly by scholarly contributions from various researchers. Hu et al. (13) have notably advanced understanding of community roles in managing intangible cultural heritage, emphasizing the necessity for community participation to achieve sustainable heritage management. Similarly, Zhang and Ala (44) highlight the importance of digital innovation in preserving sports-related intangible cultural heritage, offering critical insights into the intersection of technology and heritage management. Other scholars such as (12, 55) have explored the impacts of sports event branding on heritage tourism, emphasizing that strategic event positioning can significantly enhance tourist loyalty and regional economic performance. Boroujerdi et al. (9) have critically evaluated the positive and negative implications of sports tourism in emerging markets, underscoring the necessity of balanced development strategies that protect cultural integrity while pursuing economic growth.

Additionally (56–58), offer insights into how folklore sports enhance local identity and economic resilience within rural tourism frameworks, emphasizing the role of community-led initiatives. Scholars like Camocini et al. (59) further advocate for participatory models that integrate local community perspectives into tourism development, aligning tourism practices closely with heritage preservation. Distinct schools of thought have emerged, reflecting diverse approaches within sports tourism and heritage management discourse. The Environmental Sustainability School prioritizes ecological balance, advocating eco-friendly practices in sports tourism to mitigate adverse environmental impacts (28). The Cultural Preservation School focuses explicitly on safeguarding cultural authenticity, emphasizing stakeholder and community engagement (44). The Participatory Tourism School advocates inclusive methodologies where local communities actively shape tourism outcomes, thus ensuring cultural practices remain authentic and respected (59). In our synthesis, these contributions and schools serve as analytical categories, allowing us to identify convergences and contradictions across contexts and relate them to study quality and geographic distribution. We also interpret tensions through the authenticity–commercialization lens, linking theoretical debates to observed governance and implementation challenges.

#### 3.1.3 Debates and controversies

The integration of sports tourism and cultural heritage generates considerable scholarly debate, particularly regarding the balance between economic benefits and heritage conservation, and the tensions between authenticity and commercialization. In this review, we treat these debates as analytical lenses anchored in the Triple Bottom Line (trade-offs among economic, environmental, and socio-cultural outcomes) and Stakeholder Theory (who benefits, who bears costs, and how participation moderates impacts), and we use them to structure coding and synthesis.

Economic Benefits vs. Heritage Conservation debates hinge on the impacts of tourism-driven economic growth against potential cultural and environmental degradation. While tourism proponents highlight economic prosperity, increased job opportunities, and infrastructure improvements (10), critics emphasize the threats of over-commercialization and potential loss of cultural authenticity. Hu et al. (13) argue for the critical need to harmonize economic activities with heritage conservation, advocating participatory frameworks that integrate community perspectives in decision-making processes. In our synthesis, we identified governance safeguards (e.g., visitor caps, zoning, carrying-capacity tools, and revenue-earmarking for conservation) and implementation constraints (e.g., coordination capacity and regulatory enforcement) as moderators of this trade-off.

The Authenticity vs. Commercialization controversy revolves around maintaining genuine cultural representations amidst growing tourism demands. Yin and Lyu (56) highlight the risk of cultural dilution when local traditions become commodified for tourism. This phenomenon can transform meaningful cultural practices into superficial experiences, challenging the integrity of heritage resources. Conversely, commercialization can provide necessary funding for cultural preservation efforts, presenting a complex dilemma for tourism practitioners (28). We interpret these tensions through staged authenticity and commodification debates and code authenticity safeguards (e.g., community custodianship, ICH protocols, consent/IP arrangements, and co-created interpretation) that can balance market exposure with cultural integrity.

Overall, these theoretical frameworks, historical insights, scholarly contributions, and ongoing debates form the essential backdrop for analyzing sustainable sports tourism integrated with cultural heritage. In this study, they are used operationally—to define coding categories, interpret patterns/contradictions, and link outcomes to governance mechanisms and contextual moderators—rather than remaining descriptive background. The comprehensive understanding derived from these perspectives is critical for developing effective destination management practices that achieve balanced growth, cultural authenticity, and sustainable community benefits.

The theoretical integration model illustrates the interconnected dynamics among sustainable tourism development, the Triple Bottom Line, and stakeholder theory (Figure 3). It emphasizes balanced approaches to achieving economic growth, preserving cultural authenticity, engaging communities effectively, and ensuring environmental sustainability. Operationally, guided data extraction and synthesis: outcomes were coded to TBL domains, governance arrangements were mapped to Stakeholder Theory (e.g., co-management, partnerships), and authenticity/commercialization tensions were assessed via the presence of safeguards and implementation challenges. A high-resolution figure file is provided with the submission to ensure production-quality rendering. This integrated theoretical framework provides a comprehensive analytical tool for understanding the multifaceted dynamics of sustainable sports and heritage tourism, offering valuable insights for academics, policymakers, and practitioners striving for sustainable practices that harmonize heritage preservation, economic vitality, and community welfare.

**Figure 4:**
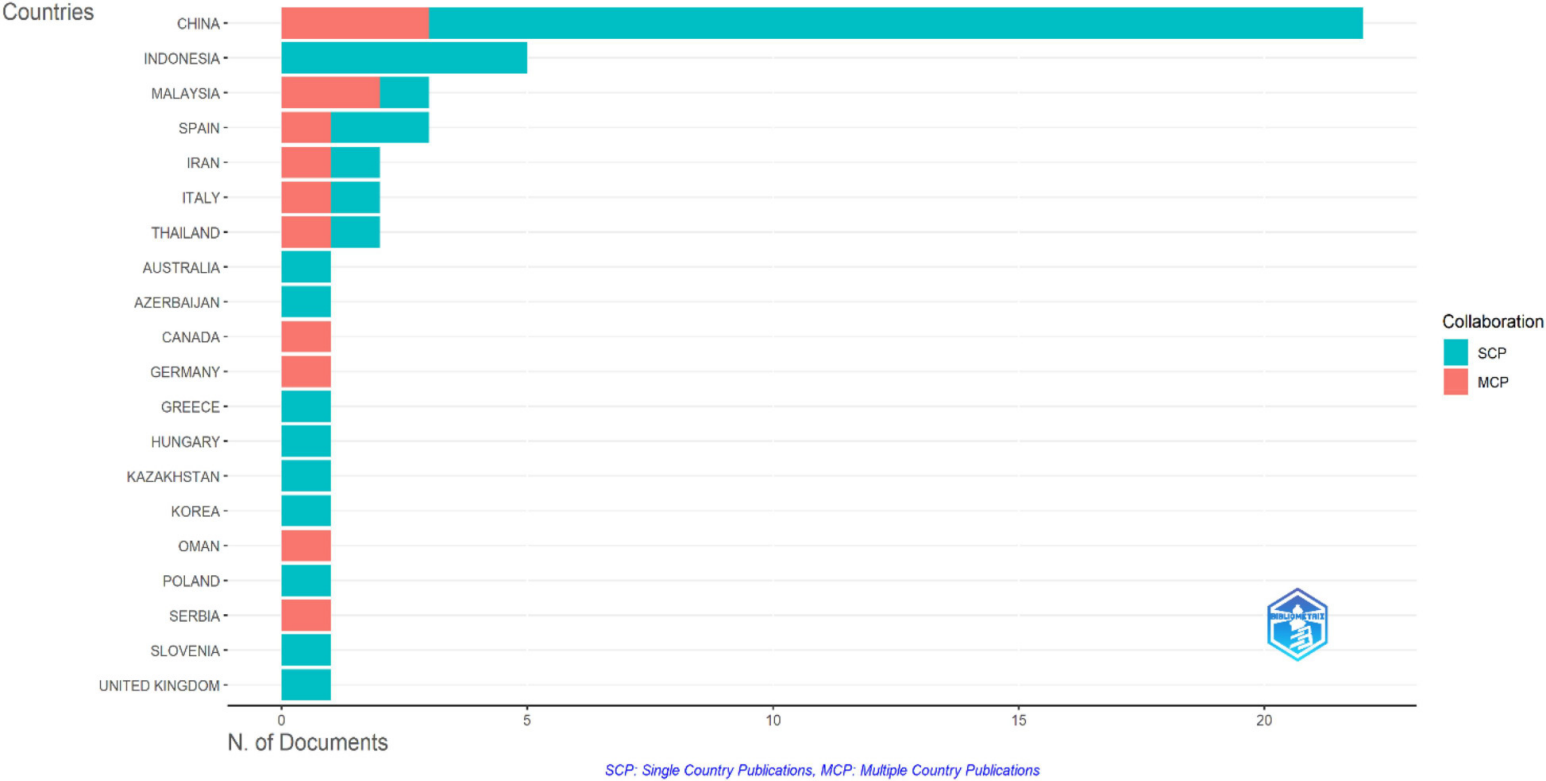
Geographic distribution.

## 4 Review of findings

### 4.1 Integration of cultural heritage in destination management

The systematic literature review highlights the critical role of cultural heritage integration within sports tourism as a strategic approach to sustainable destination management. Moving beyond description, we report a quality-weighted synthesis (MMAT/JBI) and effect-direction patterns. Studies such as those by Wang et al. (15) illustrate the successful incorporation of IoT-enabled smart experiences into Chinese Wushu heritage, significantly enhancing visitor appeal and boosting local revenue (Table 1). However, this technological integration faces challenges related to infrastructure gaps. Similarly, Li et al. (16) effectively utilized the resident-tourist emotional solidarity model in folklore sports along the Yellow River, fostering a sustainable rural tourism identity. These outcomes are moderated by context (e.g., event scale, heritage type, governance safeguards such as visitor caps and revenue-earmarking, and digital access), and regional skew is noted in Figure 4 (geographic distribution). Nonetheless, balancing the expectations of residents and tourists emerged as a key challenge.

The SWOT-led destination positioning employed by Amar et al. (17) successfully identified unique market niches in martial rituals, yet human resource deficits persisted as notable limitations. Furthermore, Yangutova et al. (18) demonstrated the effectiveness of competitiveness indices for winter heritage resorts in Siberia, although uneven infrastructure investment was highlighted as problematic. Across these cases, confidence in findings varies with study quality (reported in Supplementary Table SQA); index-based evaluations rely on proxy metrics and should be interpreted cautiously. We also observe contradictions where positioning gains are offset by workforce capacity and seasonality constraints.

Institutionalizing “karate tourism” in Okinawa by Gonzalez de la Fuente (19) successfully diversified the local economy but encountered tensions related to mainland narratives. Similarly, Kurowska et al. (20) presented landscape restoration combined with sport routes as a promising method to reduce pressure on core sites, emphasizing the need for multi-agency coordination. COVID-19 adaptations further underscored the importance of visitor flow management in UNESCO sports architecture sites, as demonstrated by Ostrowska-Tryzno and Pawlikowska-Piechotka (21). Interpreted through authenticity–commercialization debates, these cases highlight safeguards (community custodianship, ICH protocols, co-created interpretation) and implementation challenges (coordination capacity, regulatory enforcement) that shape outcomes.

### 4.2 Sustainability practices in sports tourism

The reviewed literature extensively documents sustainability practices in sports tourism, highlighting various methodologies and their outcomes (Table 2). Husain et al. (14) used an MCDA model for smart sustainable planning in Indonesia, achieving balanced quality-environmental targets, yet faced complexities in stakeholder buy-in. Jiang et al. (26) applied the TES index, pinpointing spatial hotspots needing protection but encountered significant data intensity challenges. To move beyond description, we applied a design-appropriate quality appraisal (MMAT/JBI) and synthesized results using effect-direction tallies; planning tools (e.g., MCDA, TES) generally aligned with improved targeting, but findings attenuated when lower-quality studies were excluded and when data inputs were sparse.

Tai et al. (27) introduced resource optimization through ESDA and grey correlation in the Yangtze River Delta, illustrating a shift from policy-driven to total-factor approaches, though regional disparities persisted. Furthermore, natural and socio-cultural factors notably elevated visitor satisfaction in Stojanović et al.'s (28) case study, although limited to a single reserve. Moreover, Hallmann and Zehrer (65) connected sportscapes with landscapes, significantly enhancing place identity, albeit constrained by a limited number of interviewees. We interpret these results through TBL domains: environmental gains sometimes coincided with social-equity concerns (e.g., accessibility), and capacity constraints moderated outcomes. Regional representation skews toward East Asia and alpine Europe (see geographic summary), which may limit generalizability.

### 4.3 Socio-Cultural impacts and community participation

Community engagement emerges prominently as an essential factor in managing socio-cultural impacts of sports tourism (Table 3). Marin-Pantelescu et al. (31) demonstrated that enhanced host-guest understanding could alleviate urban stress but recommended improved city liveability interventions. Li et al. (16) emphasized emotional solidarity surveys, which strengthened folklore identities but highlighted divergent resident-tourist perceptions requiring tailored engagement programs. Our quality-weighted synthesis (MMAT/JBI) indicates stronger, more consistent social outcomes where engagement mechanisms include co-management, clear benefit-sharing, and transparent communication; divergent resident–tourist perceptions are common moderators of impact.

The Tour de Ijen study by Pambudi and Hariandi (32) illustrated economic uplift and social pride but identified waste management as a persistent challenge, advocating the integration of environmental education. Similarly, ethnographic mapping by Lestari and Yusra (34) raised concerns about authenticity dilution, recommending community-led curation as a safeguard against over-commercialization. We interpret these issues through staged authenticity and commodification debates and code safeguards (e.g., community custodianship, consent/IP arrangements, cultural zoning, co-created interpretation) that balance market exposure with cultural integrity; implementation challenges (coordination capacity, waste logistics) remain recurrent.

### 4.4 Economic impacts of cultural heritage sports tourism

Economic analyses underline substantial benefits, highlighting job creation, local business support, and infrastructure development **(**summarized under Table 2, Theme B—Sustainability & Impacts, following the consolidation of tables). Synthesizing MMAT/JBI–appraised studies, we note that benefits are contingent on governance arrangements (e.g., revenue-earmarking, capacity limits) and local context. Pambudi and Hariandi (32) evidenced reduced unemployment and increased SME revenues from Tour de Banyuwangi Ijen, although waste and crowding were significant drawbacks. Despotovic and Koch (22) revealed tourism land value gains in Alpine regions, which raised critical issues concerning housing affordability and potential displacement/exclusion effects.

Chang et al. (38) utilized PLS-SEM support factors to show that resident support was crucial, although dependency risks existed. Similarly, sports tourism significantly contributed to GDP as evidenced by Lohana et al. (39), even though destination image factors showed limited moderating effects. Effects were weaker when lower-quality studies were excluded, and several models were cross-sectional, limiting causal inference; regional skew (East Asia/alpine Europe) also constrains generalizability. We therefore frame economic gains as context-dependent and recommend safeguards to mitigate affordability and dependency risks.

### 4.5 Digitalization and innovation in heritage sports tourism

Digitalization emerged as a potent driver of innovation in sports tourism (Theme C—Digital & Innovation). IoT applications, as described by Wang et al. (15), significantly enhanced visitor engagement in Wushu tourism despite existing digital divides. Big data image management significantly improved branding along the Belt and Road Initiative (60, 61, 66), although privacy, consent, and data-governance issues surfaced as critical concerns. Across MMAT/JBI–appraised studies, we find early-stage but promising evidence, moderated by access, skills, and interoperability constraints.

Qiu et al. (67) demonstrated positive emotional engagement in live-stream tourism, despite illegal content risks. Zhang and Ala's (44) implementation of ontology and NER in digital cataloguing successfully supported cultural preservation, albeit with continuous update requirements. However, most evaluations are cross-sectional or pilot-scale; effects attenuate when weighting by study quality. We therefore interpret digital gains as enabling mechanisms contingent on stakeholder capacity, ethical safeguards, and long-term maintenance funding.

### 4.6 Policy and governance in sports and heritage tourism

Policy frameworks and governance models strongly influence sustainable outcomes (Theme A—Integration & Governance; detailed rows relocated to Supplementary Table S1-Gov; main text reports a quality-weighted synthesis). Tang et al. (49) developed robust metrics through a sports-culture-tourism integration index, but evidence strength is limited by single-case validation and proxy measures. Spatial protection models proposed by Hu Q. et al. (50) effectively identified early drivers, yet regional biases remained problematic and geographic concentration limits generalizability; implementation capacity, enforcement, and coordination are recurrent constraints. We also track authenticity safeguards and commercialization risks as governance design features.

Special Protected Area planning by Sezerel and Karagoz (41) validated socio-economic triple bottom line (TBL) frameworks yet underweighted environmental indicators in practice (MMAT/JBI appraisal reduces confidence). Strategic communication models as suggested by Mazza (8) effectively shifted stakeholder attitudes, but most evaluations are cross-sectional or conceptual; we identify co-management, clear benefit-sharing, and rule enforcement as prerequisites for durable policy effects.

In summary, the systematic review identifies diverse methodologies with variable evidential strength for integrating cultural heritage within sports tourism, while highlighting key sustainability practices, socio-cultural considerations, economic impacts, digital innovations, and governance models. Theoretical frameworks such as the Triple Bottom Line and Stakeholder Theory offer useful frameworks to guide these integrations and address identified challenges, but claims are tempered by heterogeneous designs and a Scopus-only search; we provide methodologically informed recommendations rather than definitive prescriptions.

## 5 Discussion

The findings of this systematic review underline the multifaceted nature of integrating cultural heritage and sports tourism within sustainable destination management. While indicating potential synergies and context-dependent benefits, this integration is equally marked by a range of complexities and persistent challenges. This discussion explores these dimensions comprehensively, with explicit attention to methodological limitations, highlighting critical points of consideration and offering insights into future scholarly and practical pathways. Given a Scopus-only search (January 2020–June 2025) and heterogeneous study designs, findings should be interpreted as an exploratory, quality-weighted synthesis rather than definitive evidence.

Firstly, the integration of cultural heritage with sports tourism enhances visitor experiences and promotes regional identity. However, aligning traditional authenticity with contemporary tourism expectations remains challenging. Studies such as Wang et al. (15) and Li et al. (16) underscore the importance of carefully crafted event experiences, yet also highlight ongoing struggles to maintain authenticity amidst modern commercialization pressures. Interpreting these findings through the debates on *staged authenticity* and *commodification*, the tension between conserving cultural integrity and catering to tourist demands necessitates nuanced and context-sensitive management strategies, including authenticity safeguards (community custodianship, ICH protocols, co-created interpretation, and revenue-earmarking for conservation).

Secondly, participatory approaches significantly enhance stakeholder engagement and cultural resilience. Emotional solidarity frameworks and community-based models, illustrated by Li et al. (16) and Pattaray et al. (33), effectively bridge resident-tourist divides, empowering local communities. Nonetheless, variability in community readiness, resource allocation, and existing infrastructure may limit the effectiveness of such approaches. Therefore, tailored engagement strategies that align closely with local contexts and capacities are recommended to optimize community participation outcomes, with stronger effects where co-management arrangements, clear benefit-sharing, and transparent grievance mechanisms enhance legitimacy and representativeness. Practical measures include benefit-sharing agreements, capacity-building for local organizers, and accessible grievance-redress systems.

Thirdly, the implementation of sustainability frameworks, including Multi-Criteria Decision Analysis (MCDA), DPSIR models, and spatial analyses, provides valuable methodologies for sustainable tourism planning (14, 26). These methodologies facilitate informed decision-making processes but depend heavily on extensive, high-quality data and intensive stakeholder involvement. In our quality-weighted synthesis, planning tools improved targeting where robust data pipelines, monitoring, and enforcement capacity existed; no quantitative meta-analysis was attempted due to design heterogeneity. The need for comprehensive data infrastructure and collaboration underscores critical limitations, especially within resource-constrained regions, highlighting the value of standardized indicators and open data registries to support comparability and replication.

Economic impacts constitute another central area of consideration. While sports tourism offers demonstrable economic benefits—such as employment creation and SME growth—it simultaneously presents potential drawbacks, including housing affordability crises, economic over-reliance on tourism, and marginalization of traditional livelihoods (22, 43). Balancing economic development with community stability and traditional practices requires careful spatial and socio-economic planning, with attention to distributional equity (e.g., displacement risk, wage effects) and safeguards such as inclusionary zoning, event-linked earmarked tourist taxes, and local procurement commitments.

Digital innovation emerges as a critical driver for enhancing heritage sports tourism experiences, fostering both visibility and preservation of intangible heritage (13, 44). Technologies such as AI, IoT, and big data analytics significantly amplify engagement opportunities, offering new avenues for market expansion and narrative preservation. Nevertheless, issues like digital divides, privacy concerns, and gaps in regulatory frameworks remain substantial barriers. Addressing these challenges involves not only technological advancements but also robust policy frameworks and inclusive digital literacy initiatives, treating digital tools as enabling mechanisms rather than substitutes for governance and funding, and adopting privacy-by-design, consent management, and data-stewardship plans. Most evaluations are cross-sectional or pilot-scale; effects attenuate when weighting by study quality.

The preservation of cultural authenticity against commercialization pressures is a recurring debate across the reviewed literature. Authenticity often faces dilution in tourism-driven markets, raising critical concerns about the commodification of cultural assets (12, 44). Maintaining a delicate balance between cultural preservation and economic benefit thus becomes essential. Inclusive community-led curation, balanced zoning policies, and adaptive governance structures are crucial strategies to mitigate commercialization risks, situated within the frameworks of *staged authenticity* (MacCannell) and commodification debates (e.g., Greenwood). We encourage measurable authenticity indicators (community-defined integrity metrics, ICH protocol adherence) to link governance choices with outcomes.

Policy and governance structures constitute another significant dimension influencing the integration and sustainable management of sports and cultural heritage tourism. Despite several innovative policy frameworks, including Free Tourism Zones and spatial governance models (37, 50), the practical effectiveness of these models often faces limitations related to empirical validation and inter-regional coordination. Comprehensive policy strategies, supported by strategic communication (62) and participatory governance, are necessary to overcome these governance shortcomings and enhance practical outcomes, with effectiveness mediated by co-management, benefit-sharing rules, monitoring systems, and enforceability. We recommend quasi-experimental policy evaluations (pre-post with comparison sites; difference-in-differences) to strengthen causal inference about governance impacts.

Socio-cultural impacts, notably community cohesion and regional identity enhancement, emerge strongly across the reviewed literature. Sports tourism fosters social pride and economic uplift, yet simultaneously introduces challenges such as urban stress, waste management issues, and cultural commodification risks (10, 31). Addressing these socio-cultural impacts requires targeted infrastructural improvements, enhanced waste management practices, and more robust cross-sector coordination, including event environmental-management plans (waste minimization, EPR with vendors), infrastructure upgrades tied to carrying-capacity assessments, and cross-sector MOUs. Adoption of standardized socio-cultural metrics would improve comparability across cases.

Furthermore, a transdisciplinary approach is pivotal for achieving comprehensive and sustainable development in heritage sports tourism. This approach necessitates collaboration among disciplines, including economics, environmental studies, cultural heritage management, and technology sectors. The integrated, multi-dimensional analysis presented here underscores the importance of viewing sustainable tourism development (63) as inherently interdisciplinary, involving diverse stakeholders and expertise, supported by pre-registration, shared codebooks, interoperable datasets, and collaboration with community researchers.

Finally, this review identifies significant avenues for future research. Priorities include: (i) multi-database, preregistered systematic reviews with dual screening and MMAT/JBI appraisal; (ii) longitudinal and quasi-experimental impact evaluations (e.g., difference-in-differences around event rollouts); (iii) equity-focused analyses of distributional effects (housing, livelihoods); (iv) standardized authenticity indicators and cultural IP/consent protocols; (v) digital ethics frameworks and costed maintenance models; and (vi) expansion to under-represented regions (beyond East Asia/alpine Europe) via partnerships and local-language sources. Addressing these research gaps will enhance the resilience and cultural sensitivity of tourism development, ultimately contributing to sustainable community empowerment and robust destination management strategies.

## 6 Conclusion

This systematic literature review provides an exploratory, quality-weighted synthesis of the integration of cultural heritage and sports tourism within the framework of sustainable destination management (January 2020–June 2025; 63 Scopus-indexed studies). The review synthesized findings from diverse global case studies, identifying commonly reported practices in integrating cultural heritage elements in sports tourism, including robust community participation, thematic cultural events, digital innovation, and strategic destination planning. Crucially, the review reports socio-cultural and economic benefits, such as enhanced community pride, economic diversification, employment creation, and infrastructure development. However, several challenges emerged, notably maintaining cultural authenticity, preventing over-commercialization, managing environmental impacts, and balancing tourist expectations with local realities. Given the single-database (Scopus-only) search, English-language restriction, heterogeneous designs, and frequent cross-sectional evidence (precluding meta-analysis), conclusions should be interpreted with caution.

This study finds that digital technologies and innovative governance models, such as participatory governance and adaptive management frameworks, can help mitigate challenges and enhance sustainability, contingent on local capacity, data quality, and ethical safeguards (e.g., privacy-by-design, consent management). The research underscores the necessity of incorporating robust sustainability indicators, community engagement strategies, and policy frameworks aligned with the Triple Bottom Line and Stakeholder Theories. By integrating these theoretical perspectives, the findings are framed as context-dependent insights rather than universal prescriptions, specifically emphasizing cultural heritage conservation alongside economic growth.

Future research should prioritize multi-database, preregistered systematic reviews with dual independent screening and MMAT/JBI appraisal; longitudinal and quasi-experimental evaluations (e.g., difference-in-differences around event rollouts); equity-focused analyses (housing affordability, livelihood impacts, benefit-sharing); standardized authenticity indicators and ICH protocols to link governance choices with outcomes; and digital ethics frameworks with costed maintenance plans. Expanding coverage to under-represented regions and non-English sources is also recommended to reduce geographic and language bias.

Integrating cultural heritage and sports tourism offers promising, context-dependent pathways toward sustainable destination management, provided that authenticity safeguards, participatory governance, and data-enabled enforcement underpin implementation. Given the methodological constraints outlined above, these findings furnish actionable guidance while also delineating a clear agenda for cumulative, rigorous evidence-building in diverse settings.

## Data Availability

The original contributions presented in the study are included in the article/Supplementary Material, further inquiries can be directed to the corresponding author.
