# Supplementary material for: Cultural heritage and sports tourism: a systematic literature review of sustainable destination management practices
Source: Front Sports Act Living. 2025 Oct 15;7:1680229. doi: 10.3389/fspor.2025.1680229 (PMC12568658; doi:10.3389/fspor.2025.1680229)
Supplement: Supplementary file 1 [file Datasheet1.pdf]

Supplementary Material

1 Supplementary Table S0 — Inter-rater agreement (Cohen's κ)

This table reports inter-rater agreement during study screening at two stages—Title/Abstract (TA) and Full-Text (FT)—using Cohen's κ (target κ ≥ 0.70). Counts are pre-consensus; disagreements were resolved by discussion/consensus. Final post-consensus inclusion: **63 studies**.

1.1 S0. Inter-rater agreement summary

Stage	a	b	c	d	N	Po	Pe	κ	95% CI
**Title/Abstract (TA)**	120	20	24	436	600	0.927	0.639	**0.797**	To be computed in R
**Full-Text (FT)**	54	6	9	51	120	0.875	0.500	**0.750**	To be computed in R

Cell definitions. a = agree include; b = R1 include / R2 exclude; c = R1 exclude / R2 include; d = agree exclude; N = a + b + c + d.

Formulas. $Po = (a + d)/N$; $Pe = ((a + b)/N \cdot (a + c)/N) + ((c + d)/N \cdot (b + d)/N)$; $\kappa = (Po - Pe)/(1 - Pe)$.

2. Appendix A — PRISMA-S (Scopus search strategy)

**Database**	**Search string**
Scopus database	TITLE-ABS-KEY(("cultural heritage" OR "intangible cultural heritage" OR museum* OR heritage) AND ((sport* W/2 tourism) OR "sports tourism" OR "event tourism" OR marathon OR cycling OR (traditional W/1 sport*) OR (indigenous W/1 game*)) AND (sustainab* OR "destination management" OR governance OR "carrying capacity" OR "triple bottom line")) AND (PUBYEAR > 2019 AND PUBYEAR < 2026) AND (LIMIT-TO (DOCTYPE, "ar")) AND (LIMIT-TO(LANGUAGE, "English"))

For more information on Supplementary Material and for details on the different file types accepted, please see : https://www.scopus.com/results/savedList.uri?sort=plfdt-f&listId=65075695&listTypeValue=Docs&src=s&imp=t&sid=a20cd549f57a4028f4f17931826839db&sot=sl&sdt=sl&sl=0&origin=savedlist&txGid=a4f68cf939d5c3245343608c1d476933

3. Appendix B — Full-text Exclusions (PRISMA-consistent)

This appendix lists all full-text records excluded at the eligibility stage, with one primary reason code per study**, aligned with PRISMA recommendations. Counts are synchronized with the review flow:** excluded at full-text = 64**.**

Table 2.1 Full-text studies excluded and primary reason

No.	Citation	DOI/ID	Reason code	One-line reason (primary)
**1**	Y., 2022, —	10.1007/s10661-022-10584-w	E9	Wrong unit of analysis / context
**2**	A., 2022, —	10.3390/su14052553	E9	Wrong unit of analysis / context
**3**	A., 2025, —		E3	Full-text unobtainable (no DOI/ID)
**4**	H., 2023, —	10.19873/j.cnki.2096-0212.2023.02.009	E8	Outcomes not mappable (TBL/Stakeholder)
**5**	C., 2025, —	10.3390/land14030509	E9	Wrong unit of analysis / context
**6**	K., 2024, —	10.1080/15022250.2023.2202644	E1	Out of scope (no joint sport-tourism + heritage)
**7**	C., 2022, —	10.1016/j.scitotenv.2022.154122	E1	Out of scope (no joint sport-tourism + heritage)
**8**	Z., 2022, —	10.3390/land11010072	E9	Wrong unit of analysis / context
**9**	C., 2025, —	10.1007/s13132-024-02218-y	E1	Out of scope (no joint sport-tourism + heritage)
**10**	Q., 2022, —	10.5814/j.issn.1674-764x.2022.04.016	E9	Wrong unit of analysis / context
**11**	E.E., 2023, —	10.3390/f14071386	E1	Out of scope (no joint sport-tourism + heritage)
**12**	R., 2024, —	10.1057/s41599-024-03545-w	E8	Outcomes not mappable (TBL/Stakeholder)
**13**	X., 2022, —	10.1155/2022/5837919	E1	Out of scope (no joint sport-tourism + heritage)
**14**	Y., 2025, —	10.1038/s41598-025-87526-2	E9	Wrong unit of analysis / context
**15**	Y., 2022, —	10.1186/s12284-022-00577-4	E9	Wrong unit of analysis / context
**16**	M., 2023, —	10.1155/2023/9880676	E9	Wrong unit of analysis / context
**17**	D., 2021, —	10.21013/jmss.v17.n4.p8	E9	Wrong unit of analysis / context
**18**	A., 2021, —	10.47743/jopafl-2021-21-15	E9	Wrong unit of analysis / context
**19**	N., 2023, —	10.3390/su15065125	E1	Out of scope (no joint sport-tourism + heritage)
**20**	F., 2023, —	10.3389/fragi.2023.1206635	E9	Wrong unit of analysis / context
**21**	P., 2024, —	10.1016/j.heliyon.2024.e33939	E9	Wrong unit of analysis / context
**22**	B., 2025, —	10.3389/fspor.2025.1554007	E9	Wrong unit of analysis / context
**23**	A.S., 2021, —		E3	Full-text unobtainable (no DOI/ID)
**24**	I.I., 2022, —	10.5281/zenodo.5980129	E1	Out of scope (no joint sport-tourism + heritage)
**25**	W., 2024, —	10.1016/j.jenvman.2024.120554	E9	Wrong unit of analysis / context
**26**	Y., 2024, —	10.47197/retos.v61.108284	E9	Wrong unit of analysis / context
**27**	A., 2024, —	10.14505/tpref.v15.2(30).11	E9	Wrong unit of analysis / context
**28**	R.J., 2022, —	10.3390/su14031232	E1	Out of scope (no joint sport-tourism + heritage)
**29**	K., 2024, —	10.57239/pjlss-2024-22.2.00637	E9	Wrong unit of analysis / context
**30**	P., 2023, —		E3	Full-text unobtainable (no DOI/ID)

**31**	Q., 2024, —	10.1016/j.heliyon.2024.e38689	E9	Wrong unit of analysis / context
**32**	P., 2025, —	10.3390/su17114995	E1	Out of scope (no joint sport-tourism + heritage)
**33**	B., 2023, —	10.3390/su1509703	E1	Out of scope (no joint sport-tourism + heritage)
**34**	L., 2022, —	10.3390/su141810990	E9	Wrong unit of analysis / context
**35**	H., 2022, —	10.20944/preprints202209.0445.v1	E2	Not a peer-reviewed Article
**36**	U., 2022, —	10.17509/jpjo.v7i1.39375	E9	Wrong unit of analysis / context
**37**	L., 2024, —	10.3390/hospitality4020017	E9	Wrong unit of analysis / context
**38**	Z., 2024, —	10.3390/land13020258	E9	Wrong unit of analysis / context
**39**	F., 2024, —	10.32407/att.1402.07	E1	Out of scope (no joint sport-tourism + heritage)
**40**	O., 2023, —	10.3390/ijerph2020748	E9	Wrong unit of analysis / context
**41**	B., 2022, —	10.20944/preprints202210.0468.v1	E2	Not a peer-reviewed Article
**42**	H., 2023, —	10.3389/fenvs.2023.1190582	E9	Wrong unit of analysis / context
**43**	A., 2023, —	10.1016/j.apenergy.2023.122709	E9	Wrong unit of analysis / context
**44**	T., 2023, —	10.1016/j.jksus.2023.102640	E9	Wrong unit of analysis / context
**45**	R., 2023, —	10.1080/14616688.2023.2228117	E9	Wrong unit of analysis / context
**46**	B., 2023, —	10.7910/dvn/1z3sym	E2	Not a peer-reviewed Article
**47**	K., 2022, —	10.1371/journal.pone.0266622	E9	Wrong unit of analysis / context
**48**	M., 2024, —	10.3390/su16020873	E1	Out of scope (no joint sport-tourism + heritage)
**49**	P., 2024, —	10.1016/j.dib.2024.110713	E2	Not a peer-reviewed Article
**50**	Y., 2022, —	10.1080/19388160.2021.1975005	E1	Out of scope (no joint sport-tourism + heritage)
**51**	Y., 2024, —	10.2478/amns-2024-0548	E9	Wrong unit of analysis / context
**52**	K., 2022, —	10.30892/gtg.454spl02-973	E1	Out of scope (no joint sport-tourism + heritage)
**53**	K., 2022, —	10.1177/22338659221120973	E9	Wrong unit of analysis / context
**54**	P., 2024, —	10.3233/jifs-230547	E9	Wrong unit of analysis / context
**55**	S., 2023, —	10.1016/j.heliyon.2023.e21732	E9	Wrong unit of analysis / context
**56**	Z.H., 2025, —	10.59429/esp.v10i5.3700	E9	Wrong unit of analysis / context
**57**	M.S., 2024, —	10.16926/sit.2024.03.08	E1	Out of scope (no joint sport-tourism + heritage)
**58**	D., 2022, —	10.20867/thm.28.3.15	E9	Wrong unit of analysis / context
**59**	Husain, 2024, —	10.18280/mmep.111116	E9	Wrong unit of analysis / context
**60**	N.Zh., 2024, —	10.30892/gtg.53216-1228	E1	Out of scope (no joint sport-tourism + heritage)
**61**	E., 2022, —	10.30892/gtg.44407-939	E9	Wrong unit of analysis / context
**62**	S.S., 2023, —	10.2478/pjst-2023-0010	E1	Out of scope (no joint sport-tourism + heritage)
**63**	P., 2022, —	10.3390/land11101667	E9	Wrong unit of analysis / context

**64**	R., 2025, —	10.61091/jcmcc127a-407	E1	Out of scope (no joint sport-tourism + heritage)

Reason codes (choose exactly one per study)

- **E1 Out of scope.** Does not substantively address both *sport tourism* and *cultural heritage*/ICH in the same analysis.
- **E2 Not a peer-reviewed article.** Editorial, commentary, conference paper, book/chapter, etc. *(Should be 0 here if filtered at search stage.)*
- **E3 Full-text not available.** Could not obtain full text after reasonable attempts.
- **E4 Inadequate design/methods.** Design cannot address the review question (e.g., missing empirical basis, unclear sampling).
- **E5 Outside year range.** Publication year beyond pre-specified limits. *(Should be 0 here if filtered at search stage.)*
- **E6 Outside language criteria.** Not in included language(s). *(Should be 0 here if filtered at search stage.)*
- **E7 Duplicate at FT stage.** Residual duplicates detected when inspecting full texts.
- **E8 Irrelevant outcomes.** Outcomes cannot be mapped to the TBL/Stakeholder framework.
- **E9 Wrong unit of analysis.** Not a relevant context (e.g., not a destination/activity; purely conceptual without empirical focus where empirical evidence is required).
- **E10 Other (specify).** Provide a one-line specific description.

2 Supplementary Figures and Tables

2.1 Suplementary Tabel

2.2 Quality Appraisal Table

Table SQA 1. (Condensed—Theme A: Integration & Governance)

Full study details are provided in Supplementary Table S1. This main-text table retains headline rows only, which illustrate key cross-study patterns and contradictions. We also report effect-direction tallies and indicate where findings attenuate after excluding lower-quality studies.

No	Author(s) & Year	Region / UN Subregion	Design	Quality Score (MMAT/JBI)	Cultural Heritage Aspects	Integration Methods	Effectiveness (Headline Finding)	Authenticity Safeguards / Implementation Challenges

1	Wang et al., 2023	Eastern Asia / China	Quantitative	MMAT-3	Chinese Wushu heritage	IoT-enabled smart experiences; event co-branding	Boosted visitor appeal & local revenue	Tech infrastructure gaps; data integration needs
2	Li et al., 2025	Eastern Asia / China	Mixed-method	JBI-4	Folklore sports along Yellow River	Resident–tourist emotional solidarity model	Enhanced identity & sustainable tourism	Expectation balancing between locals and tourists
3	Amar et al., 2020	South-Eastern Asia / Indonesia	Qualitative	JBI-3	Taji Tuta martial ritual	SWOT-led destination positioning	Identified unique market niche	Local HR limitations; strategic alignment gaps
4	Yangutova et al., 2023	Northern Asia / Russia	Quantitative	MMAT-4	Siberian ski-landscape traditions	Competitiveness index for heritage winter resorts	Flagged Sobolinaya as prime hub	Uneven investment; governance asymmetry
5	Gonzalez de la Fuente, 2021	Eastern Asia / Japan	Qualitative	JBI-3	Karate heritage in Okinawa	Institutionalisation of "karate tourism"	Diversifies local economy	Tensions with national narratives; cultural filtering
6	Kurowska et al., 2023	Eastern Europe / Poland	Mixed-method	MMAT-3	Historic quarries & forest trails	Landscape restoration + sport routes	Reduced pressure on core sites	Multi-agency coordination complexity

7	Ostrowska-Tryzno & Pawlikowska-Piechotka, 2021	Eastern Europe / Poland	Case study	JBI-4	UNESCO sports architecture	Re-designing visitor flows post-COVID	Maintained cultural magnetism safely	Sanitary compliance cost; tourism safety perceptions
8	Du et al., 2025	Eastern Asia / China	Quantitative	MMAT-3	Tulou World Heritage marathon	Event-heritage image coupling	Raised tourist loyalty	Weak place-attachment; low post-event engagement
9	Despotovic & Koch, 2025	Southern Europe / Alps	Quantitative	MMAT-4	Alpine heritage landscapes	Spatial econometrics (land value vs ski culture)	Showed heritage premium	Housing affordability trade-offs; local pushback
10	Echeverri et al., 2025	South America / Colombia	Mixed-method	JBI-4	Colombian biocultural richness	Integrated biodiversity-culture mapping	Identified untapped destinations	Site inaccessibility; need for conservation skills
11	de Freitas et al., 2025	Southern Europe / Portugal	Quantitative	MMAT-4	Portuguese medieval castles	Quantile regressions on tourism flows	Castles extend visitor stay	Holistic rural packages needed; disjointed stakeholder roles

Table SQA 2. Sustainability and Impacts in Heritage Sports Tourism (Condensed—Theme B)
Detailed per-study findings are provided in Supplementary Table S2. This main-text table synthesizes cross-study patterns, contradictions, and sensitivity to study quality. Regional representation, study design, and quality indicators are included to highlight the scope, strengths, and limitations of the literature.

No	Author(s) & Year	Region / UN Subregion	Design	Quality Score (MMAT/JBI)	Sustainability Measures	Outcomes	Implementation Constraints / Challenges
**1**	Husain et al., 2024	South-Eastern Asia / Indonesia	Quantitative	MMAT-4	MCDA model for smart sustainable planning	Balanced quality–environment targets	Complex stakeholder buy-in
**2**	Jiang et al., 2025	Eastern Asia / China	Mixed-method	MMAT-3	TES index (DPSIR)	Spatial hotspots for protection	High data intensity; replicability issues
**3**	Tai et al., 2024	Eastern Asia / China	Mixed-method	MMAT-3	ESDA + grey correlation analysis	Shift to total factor-driven tourism	Regional disparities in resource allocation
**4**	Stojanović et al., 2024	Southern Europe / Serbia	Quantitative	JBI-3	Prism of Sustainability survey	Natural & sociocultural factors raise satisfaction	Sample limited to a single reserve
**5**	Zhensikbayeva et al., 2024	Central Asia / Kazakhstan	Mixed-method	JBI-3	GIS-based resource visualization	Thematic sport-tourism routes proposed	Mountain data scarcity; mapping limitations
**6**	Hallmann & Zehrer, 2024	Western Europe / Alps	Qualitative	JBI-4	Sportscape–landscape integration	Sportscape enhances place identity	Small sample size; interpretive generalizability
**7**	Boroujerdi et al., 2023	Western Asia / Iran	Mixed-method	MMAT-3	MICMAC analysis of	Prioritization for sustainable	Institutional instability in

					critical factors	governance	emerging market
**8**	Fu & Liang, 2020	Eastern Asia / China	Case study	JBI-2	SWOT audit of island leisure sites	Unique marine appeal emphasized	Pre-COVID data; current applicability limitations
**9**	Kurowska et al., 2023	Eastern Europe / Poland	Case study	JBI-3	Forest quarry re-purposing	Reduced trail pressure; nature preservation	Limited restoration funding; scale-up issues
**10**	Hui et al., 2022	Eastern Asia / China	Quantitative	MMAT-3	Top 5 environmental indicator ranking	Orderliness key for eco-sport experiences	Survey-based data; lack of longitudinal depth
**11**	Hu et al., 2024	Eastern Asia / China	Mixed-method	JBI-4	IoT-based tourism coupling model	Tourism–sport synergy evidenced	Requires longitudinal validation; tech adoption gap

Note: (Table body relocated to Supplementary Table S2. Headline rows retained in the main text only where they inform cross-study patterns and contradictions. We report effect-direction tallies within this theme and indicate where results attenuate after excluding lower-quality studies. No meta-analysis was conducted due to design heterogeneity.)

Table SQA 3. Theme C—Socio-cultural & Participation (Condensed)
(Detailed per-study rows relocated to Supplementary Table S3. The main text synthesizes cross-study patterns, conflicts, and quality-sensitivity; we also summarize regional representation to clarify global versus regional perspectives.)

No	Author(s) & Year	Region / UN Subregion	Design	Quality Score (MMAT/JBI)	Engagement Mechanism	Positive Impacts	Negative Impacts	Authenticity Safeguards / Recommendations

**1**	Marin Pantelescu et al., 2022	Europe / Eastern Europe	Focus Group Discussions (FGDs)	Moderate	Focus groups with Erasmus students	Enhanced host–guest understanding	Urban stress reported	Improve city livability and tourist flow management
**2**	Li et al., 2025	Asia / East Asia	Survey-based analysis	High	Emotional solidarity surveys	Stronger folklore identity	Divergent resident vs tourist views	Tailored engagement and communication programs
**3**	Pambudi & Hariandi, 2021	Asia / Southeast Asia	Multi-stakeholder interviews	Moderate	Tour de Ijen stakeholder discussions	Economic uplift, social pride	Waste management strain	Integrate environmental education in event planning
**4**	Mair et al., 2023	Global	Narrative SLR	High	Review of mega event narratives	Framework for social benefit analysis	Measurement inconsistency	Standardize metrics for cross-case evaluations
**5**	Widianingsih et al., 2023	Asia / Southeast Asia	Digital media content analysis	Moderate	Digital trend analysis (F1H2O)	Regional branding via online promotion	Event preparation issues	HR upskilling, coordinated logistics
**6**	Pattaray et al., 2025	Asia / Southeast Asia	FGDs + community planning	High	MotoGP participatory workshops	HR development framework	Risk of cultural commodification	Inclusive capacity building with local actors
**7**	Stojanović et al., 2024	Europe / Southern Europe	Resident and visitor surveys	High	Visitor feedback on natural sites	High satisfaction with nature & culture	Environmental concerns under-communicated	Embed sustainability themes in communication

**8**	Lestari & Yusra, 2022	Asia / Southeast Asia	Ethnographic mapping	High	Sasak traditional practice exploration	New ethno-attractions catalogued	Fear of authenticity dilution	Ensure community-led heritage curation
**9**	Wen, 2023	Asia / East Asia	Spatial diffusion analysis	Moderate	Mapping ethnic sport geography	Cultural landscape integration	Over-commercialization	Cultural zoning and stakeholder consultation
**10**	Komaini et al., 2025	Asia / Southeast Asia	Participatory rural appraisal	High	Village-level participatory study	Boost to local economy via sports tourism	Stakeholder coordination gaps	Establish cross-sector collaboration channels
**11**	Usmanova et al., 2020	Asia / Central Asia	Policy conceptual model	Moderate	Concept of free tourism zones	Unlocking heritage tourism potential	Low baseline of tourist flow	Invest in infrastructure and targeted marketing

Table S2-Econ (Theme B—Sustainability & Impacts, Economic Focus, Condensed)
(Detailed per-study rows relocated to Supplementary Table S2-Econ. The consolidated main-text table adds **Design**, **Region/UN subregion**, **Quality score (MMAT/JBI)**, and **Equity/Safeguards** columns; results are synthesized via effect-direction tallies. No meta-analysis was conducted due to design heterogeneity.)

**No**	**Author(s) & Year**	**Event/Activity**	**Economic Metrics**	**Positive Impacts**	**Negative Impacts**	**Region / UN Subregion**	**Design**	**Quality Score (MMAT/JBI)**	**Equity / Safeguards**
**1**	Pambudi & Hariandi, 2021	Tour de Banyuwangi Ijen	Ticket sales ↑ 100%; SMEs revenue +43%	Reduced unemployment	Waste & crowding	Southeast Asia / Indonesia	Mixed methods	High	Integrate environmental education

**2**	Despotovic & Koch, 2025	Alpine land price model	Spatial Durbin model – ski premium	Increased land value	Housing affordability	Europe / Western Europe	Quantitative (modeling)	Medium	Regional zoning policies
**3**	Chang et al., 2020	Sport tourism dependency	PLS SEM support factors	Resident support for sports projects	Dependency risk	East Asia / Taiwan	Quantitative (SEM)	High	Diversification strategy
**4**	Lohana et al., 2023	Mediation moderation model	SEM linking env/culture & economy	GDP growth driven by sports tourism	Destination image not moderating	South Asia / Pakistan	Quantitative (SEM)	High	Enhance tourism branding
**5**	Sarmento & Monteiro, 2023	Tarrafal hub workshops	Stakeholder SWOT	Diversification of local economy	Infrastructure upgrades needed	Africa / West Africa	Qualitative	Medium	Multi-sector investment strategy
**6**	Sezerel & Karagoz, 2023	Datça SPA surveys	Economic vs environmental exchange	Local economic support	Environmental impacts undervalued	Europe / Southern Europe	Quantitative (survey)	Medium	Strengthen env. valuation in planning
**7**	Dirin et al., 2023	Todzhinsky district GIS	Tourism potential mapping	Investment clustering identified	Limited access routes	Asia / Russia	GIS-based mixed methods	High	Rural connectivity plans
**8**	Zhang et al., 2024	West Sichuan integration index	TOPSIS rankings	Education investments	Inter-regional inequalities	East Asia / China	Quantitative (MCDM)	High	Targeted social investment

				increased					
**9**	Offenhenden & Soronellas, 2021	Pyrenees ski vs farming	Economic case study	Rural income supplemented	Agriculture marginalization	Europe / Southern Europe	Qualitative case study	Medium	Diversify rural livelihood programs
**10**	Ma et al., 2024	Sports fitness rural tourism	Governance & satisfaction scores	Economic & environmental gains	Overuse risks	East Asia / China	Mixed methods	High	Visitor capacity control

Table 3. Theme C — Digital & Innovation (Condensed Summary)
Main-text table includes Design, Region/UN Subregion, Quality score (MMAT/JBI), Ethics/data governance, and Scalability columns. Detailed entries are located in Supplementary cc. Synthesis emphasizes key cross-study patterns, contradictions, and links to Stakeholder Theory (risk/benefit equity) and Triple Bottom Line outcomes.

No	Author(s) & Year	Technology / Innovation	Region / UN Subregion	Design	Quality Score (MMAT/JBI)	Ethics / Data Governance	Scalability Potential	Outcomes	Challenges
**1**	Wang et al., 2023	IoT for Wushu tourism	China / Eastern Asia	Mixed Methods	High	Data privacy policies unclear	Medium	Enhanced engagement	Digital divide
**2**	Cao & Xiao, 2024	AI big data image management	China / Eastern Asia	Quantitative	Moderate	UGC regulation required	High	Improved destination branding	Data privacy
**3**	Qiu et al., 2021	Live stream tourism	China / Eastern Asia	Content Analysis	Moderate	Weak filtering systems	Medium	Positive tourist emotions dominate	Illegal content risk

**4**	Sun et al., 2022	Fuzzy analysis for competitiveness	China / Eastern Asia	Quantitative	High	Transparent algorithm ethics	Medium	Enhanced benchmarking for destinations	Model complexity
**5**	Zhang & Ala, 2024	Ontology & NER for ICH	China / Eastern Asia	Qualitative	High	Data integrity assurance	Medium	Digital preservation support	Continual updates needed
**6**	Hao et al., 2024	Big data sentiment mining (CF tree)	China / Eastern Asia	Quantitative	Moderate	Anonymity in clustering required	High	Gender & age-based segmentation insights	Short trip dominance
**7**	Hong et al., 2022	AI-enabled sport-tourism coupling	China / Eastern Asia	Statistical Modeling	High	AI interpretability challenge	Low	Innovative sport-culture mapping	Incomplete data
**8**	Hu et al., 2024	IoT grey relational analysis	China / Eastern Asia	Quantitative	High	Centralized system governance	High	Strong coupling: sport & tourism synergy	Platform scalability
**9**	Ieong, 2024	SWOT analysis for Macao study tours	Macao / Eastern Asia	Qualitative	Moderate	SWOT data ethics implicit	Medium	Leveraged National Games hype	Policy framework gaps
**10**	Wu et al., 2024	Athlete-centered night tourism analytics	China / Eastern Asia	Mixed Methods	Moderate	Consent for nighttime profiling	High	Strategic growth opportunities	Attention competition

11	Gharibzadeh et al., 2023	Grounded theory on sports tours	Iran / Southern Asia	Qualitative	High	Participant consent enforced	Medium	Mapped facilitators vs inhibitors	Financial & security barriers

Table SQA 1. Theme A — Integration & Governance (Condensed Summary)

No	Author(s) & Year	Policy Framework	Governance Model	Region/UN Subregion	Design	Quality Score (MMAT/JBI)	Strengths	Limitations	Implementation Challenges	Authenticity Safeguards
1	Tang et al., 2025	Sports–Culture–Tourism integration index	Event venue evaluation	East Asia (China)	Quantitative	High	Reliable integration metrics	Single case validation	Scaling integration frameworks	Localized stakeholder feedback
2	Hu Q. et al., 2024	Spatial ICH protection model	Geodetector-based	East Asia (China)	Quant-Qual	High	Early detection of cultural drivers	Regional (eastern) bias	Limited application in rural zones	Participatory mapping
3	Sezerel & Karagoz, 2023	Special Protected Area planning	Mixed-method resident input	Western Asia (Turkey)	Mixed	Moderate	SET/TBL model validation	Environmental impacts undervalued	Resident alignment with developers	Environmental ethics training
4	Usmanova et al., 2020	Free Tourism Zones law	National strategic zoning	Central Asia (Uzbekistan)	Qualitative	Moderate	Legal foundation for cultural	Infrastructure deficits	Governance enforcement issues	Local cultural codes

							unlocking			
**5**	Kaur et al., 2023	Fuzzy LP marketing allocation	State budgeting	South Asia (India)	Quantitative	Moderate	High ROI potential	Hypothetical scenario	Need real-time tourism data	Heritage benchmarking
**6**	Akhundova, 2024	Festival development strategy	Functional analysis	Western Asia (Azerbaijan)	Qual-Quant	High	Regional branding enhancement	Generic festival typology	Cultural specificity dilution	Folklore representation standards
**7**	Mazza, 2023	Strategic communication model	Stakeholder engagement	Europe (Italy)	Conceptual	Low	Behavior change orientation	Needs empirical validation	Sustained dialogue with all actors	Inclusive participation protocols
**8**	Li L., 2025	Cross-regional value transmission	Philosophical synthesis	East Asia (China)	Conceptual	Low	Innovative transregional thinking	Lacks measurable metrics	Difficult real-world application	Narratives respecting local culture
